# Allergic Contact Dermatitis From Norway Spruce Resin in a Wound Ointment (Abilar)—A Case Suggesting Primary Sensitization

**DOI:** 10.1111/cod.70103

**Published:** 2026-02-05

**Authors:** Kevin Yang, Cecilia Svedman, Thanisorn Sukakul

**Affiliations:** ^1^ Department of Dermatology, Faculty of Medicine, Lund University Skåne University Hospital Lund Sweden; ^2^ Department of Occupational and Environmental Dermatology, Faculty of Medicine, Lund University Skåne University Hospital Malmö Sweden

**Keywords:** colophonium, contact allergy, medical device, Norway spruce, repeated open application test, scar, wound

We describe a case of allergic contact dermatitis (ACD) to Abilar (Repolar Pharmaceuticals Oy, Espoo, Finland), a wound ointment containing *Picea abies* (Norway spruce) resin with evidence suggesting primary sensitization.

## 1 Case Report

A 38‐year‐old non‐atopic woman presented with a localised eczema which developed 10 days after applying Abilar wound ointment and Xylocaine cream, both recommended by the pharmacy, on a superficial burn area on her arm for pain relief and to prevent scarring. The patient denied any previous skin problems or eczema suggesting contact allergy to any other products or substances prior to this incident.

**FIGURE 1:**
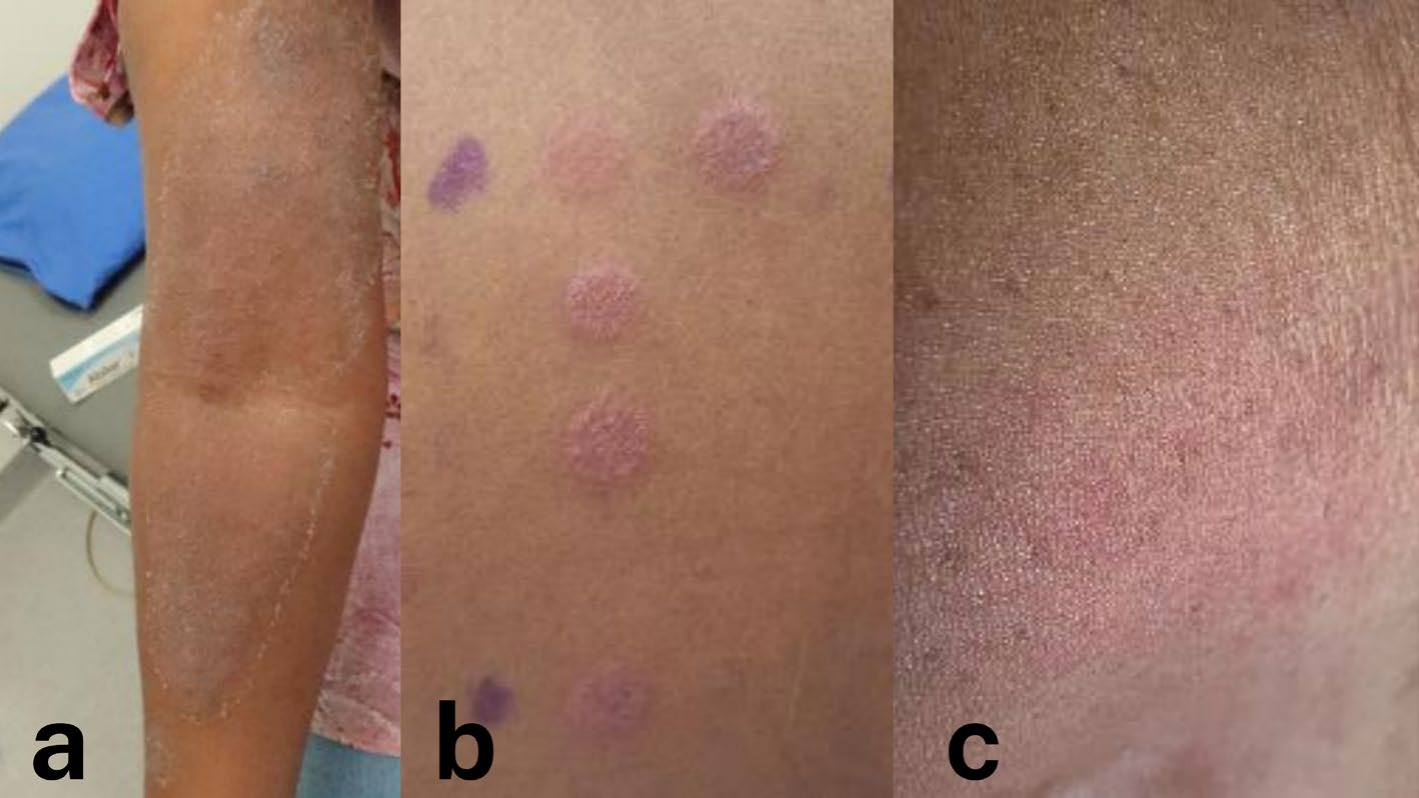
Clinical presentations, patch test reactions and repeated open application test result in the patient. (a) Local reaction after using Abilar for 10 days. (b) Patch test with strong positive reactions to Abilar. (c) A positive repeated open application test after 1 day of using Abilar.

Patch testing was performed using the Swedish and Malmö extended baseline series, caine series, patient's own products, product excipients and additional resin‐related substances. Strong positive reactions were observed to Abilar, colophonium and other related substances (Table 1). A repeated open application test (ROAT) with Abilar was performed on intact skin on the volar arm which induced an eczematous reaction after 1 day and worsened with continued application. Figure 1 illustrates the patient's clinical presentation, patch test reactions and the result of the ROAT.

**TABLE 1:** Patch test results.

Test series and preparation	Concentration and vehicle	Result^a^
Baseline series		
Colophonium	20% pet.	++
*Myroxylon pereirae* resin	25% pet.	+
Textile dye mix	6.6% pet.	+
Hydroperoxides of limonene	0.3% pet.	+
Other test preparations in the series		−
Caine series		
All test preparations		−
Personal products		
Abilar wound ointment	as is	++
Xylocaine cream	as is	−
Other test preparations		
Abietic acid	10% pet.	++
Hydroabietyl alcohol	10% pet.	+
Canada balsam	25% pet.	++
Glyceryl hydrogenated rosinate	20% pet.	+
Methyl hydrogenated rosinate	20% pet.	+
Sorbitan oleate	5% pet.	−

*Note:* Pet, petrolatum; ++, strong positive reaction; +, weak positive reaction; −, negative reaction.

^a^ Strongest reactions on Day 3/4 or 7 are reported.

## 2 Discussion

Abilar is a prescription‐free medical device containing 10% Norway spruce resin, marketed as a topical product for several skin conditions including those with skin barrier defects. Few studies regarding whether Abilar has potential to induce sensitization and elicit ACD have been published [1, 2]. Previously reported cases with ACD to Abilar showed a similar pattern of contact allergies, that is, to colophonium‐ and fragrance‐related substances [1]. Both Norway spruce resin and colophonium are complex natural mixtures with similar chemical profiles. Consequently, patients sensitised to Norway spruce resin may also react to colophonium‐related substances and are at risk of developing contact dermatitis from medical devices containing these resins.

In another study, no sensitization occurred after 21 days of exposure to Abilar on healthy skin [2]. However, as sensitization to medical devices may take months to develop even under occlusion [3], short‐term exposure on undamaged skin may underestimate the true sensitization potential.

In our case, a patient without prior skin problems had initially tolerated Abilar but developed symptoms after 10 days. Subsequent ROAT demonstrated rapid elicitation. These findings suggest that Abilar can not only elicit reactions but also induce de novo sensitization, particularly on damaged skin. The patient was simultaneously advised to use topical Xylocaine cream, and considering the impaired skin barrier and confirmed sensitization, it is fortunate that she did not also become sensitised to lidocaine.

In conclusion, it is of clinical importance to recognise that Abilar can induce contact allergy. Its sensitising potential should be clearly highlighted to distributors, caregivers and patients to prevent potentially harmful use on compromised skin.

## Author Contributions

**Kevin Yang:** conceptualization, investigation, writing – original draft, writing – review and editing. **Cecilia Svedman:** supervision, conceptualization, investigation, writing – review and editing. **Thanisorn Sukakul:** conceptualization, investigation, supervision, writing – review and editing.

## Funding

The authors have nothing to report.

## Conflicts of Interest

The authors declare no conflicts of interest.

## Data Availability

The data that support the findings of this study are available from the corresponding author upon reasonable request.
